# Longitudinal Lipid Trajectories and Progression of CKD in Children

**DOI:** 10.1016/j.ekir.2025.02.007

**Published:** 2025-02-17

**Authors:** Uwe Querfeld, Marietta Kirchner, Francesca Mencarelli, Karolis Azukaitis, Aysun Bayazit, Ali Duzova, Anke Doyon, Nur Canpolat, Ipek Kaplan Bulut, Lukasz Obrycki, Justine Bacchetta, Rukshana Shroff, Dusan Paripovic, Cengiz Candan, Jerome Harambat, Alev Yilmaz, Harika Alpay, Jun Oh, Hakan Erdogan, Claus P. Schmitt, Anette Melk, Franz Schaefer

**Affiliations:** 1 Department of Pediatric Gastroenterology, Nephrology and Metabolic Diseases, Charité University Hospital, Berlin, Germany; 2 Institute for Medical Biometry and Informatics, University of Heidelberg, Heidelberg, Germany; 3 Pediatric Nephrology Unit, Department of Pediatrics, S. Orsola-Malpighi Hospital, University of Bologna, Bologna, Italy; 4 Clinic of Pediatrics, Institute of Clinical Medicine, Faculty of Medicine, Vilnius University, Vilnius, Lithuania; 5 Department of Pediatric Nephrology, Cukurova University, Adana, Turkey; 6 Division of Pediatric Nephrology, Hacettepe University Faculty of Medicine, Ankara, Turkey; 7 Pediatric Nephrology Division, Department of Pediatrics I, University of Heidelberg, Heidelberg, Germany; 8 Department of Pediatric Nephrology, Istanbul University Cerrahpasa Faculty of Medicine, Istanbul, Turkey; 9 Division of Pediatric Nephrology, Department of Pediatrics, Ege University Faculty of Medicine, Izmir, Turkey; 10 Department of Nephrology, Kidney Transplantation and Hypertension, Children’s Memorial Health Institute, Warsaw, Poland; 11 Pediatric Nephrology Unit, Hôpital Femme Mère Enfant, Hospices Civils de Lyon, Université de Lyon, Lyon, France; 12 UCL Great Ormond Street Institute of Child Health, London, UK; 13 Nephrology Department, University Children’s Hospital and School of Medicine, University of Belgrade, Serbia; 14 Division of Pediatric Nephrology, Istanbul Medeniyet University, Göztepe Hospital, Istanbul, Turkey; 15 Pediatric Nephrology Unit, Department of Pediatrics, Bordeaux University Hospital, France; 16 Istanbul University Istanbul Faculty of Medicine, Istanbul, Turkey; 17 Department of Pediatric Nephrology, Marmara University Faculty of Medicine, Istanbul, Turkey; 18 Pediatric Nephrology, UKE University Children's Hospital, Hamburg, Germany; 19 Department of Pediatric Nephrology, Dörtçelik Children’s Hospital, Bursa, Turkey; 20 Department of Kidney, Liver and Metabolic Diseases, Hannover Medical School, Hannover, Germany

**Keywords:** children, chronic kidney disease, dyslipidemia, progression, proteinuria

## Abstract

**Introduction:**

There are discrepant findings regarding the effect of dyslipidemia on disease progression in adult patients with chronic kidney disease (CKD).

**Methods:**

In a prospective cohort study of children with stage 3 to 5 (predialysis) CKD, triglycerides (TGs), total cholesterol (CHOL), low-density lipoprotein cholesterol (LDL-C), and high-density lipoprotein cholesterol (HDL-C) were measured semiannually. We investigated whether CKD progression is associated with serum lipid levels at baseline and with lipid trajectories during follow-up. CKD progression was defined as the time to a composite event of 50% reduction in estimated glomerular filtration rate (eGFR), eGFR < 10 ml/min per 1.73 m^2^, or start of kidney replacement therapy. By semiparametric group-based trajectory modeling (GBTM), 2 trajectories were defined for each lipid, termed “high” and “low.”

**Results:**

A total of 681 patients aged 12.2 ± 3.3 years with a mean eGFR of 26.9 ± 11.6 ml/min per 1.73 m^2^ were included. Kidney diagnosis was classified as congenital anomalies of the kidneys and urinary tracts (CAKUT) in 69%, glomerulopathy in 8.4%, and other disorders in 22.6% of patients. During a median of 5.1 years of follow-up, 59% of patients reached the composite end point. Kidney survival was significantly different for HDL-C (*P* = 0.0128), but not for other lipid trajectories in the Kaplan-Meier analysis. There was no significant association of any of the lipid trajectories with CKD progression in Cox proportional hazard models. Variables consistently associated with CKD progression in models for each lipid at baseline and for lipid trajectories included age, a diagnosis other than CAKUT, eGFR at baseline, albuminuria, the serum albumin level, and diastolic blood pressure (BP).

**Conclusions:**

These data do not support an important role for lipids in the progression of CKD in children.

## Graphical abstract

**Figure:**
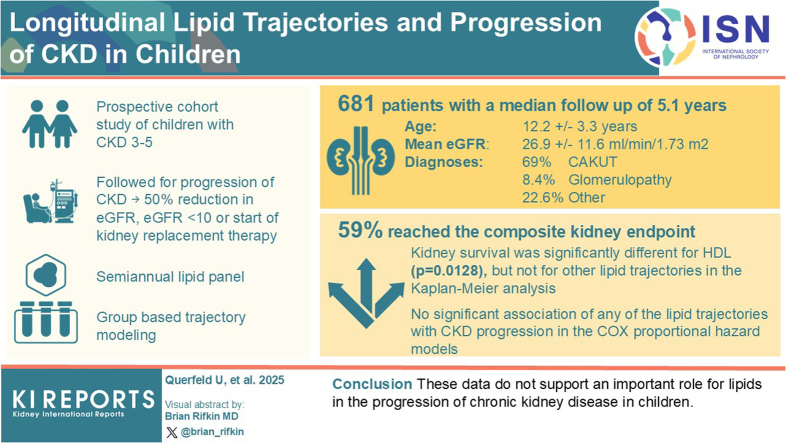

See Commentary on Page 1324

CKD is accompanied with profound changes in the composition of lipoproteins and their levels in serum. The typical pattern of “uremic dyslipidemia” is characterized by increased levels of TGs and a decrease in HDL-C, whereas levels of CHOL and LDL-C are variable.^1^ Patients with nephrotic syndrome or nephrotic range proteinuria have highly elevated levels of CHOL and LDL-C.^2^^,^^3^ Apart from proteinuria, a variety of factors have modifying effects on the lipid profile, such as primary kidney disease, CKD stage, medications, diet, the presence of malnutrition or obesity, and sex-specific effects.^4^^,^^5^

In the adult general population, dyslipidemia is not only a traditional risk factor for atherosclerosis and cardiovascular disease, but also, most often in the form of high TGs and/or low HDL-C, for the incidence of new-onset CKD.^6^, ^7^, ^8^, ^9^, ^10^ In line with these observations, dietary lipids cause kidney injury in several animal models,^11^ which can be ameliorated by cholesterol-lowering medications.^12^ Although these population studies and experimental data in animal models of CKD suggest an impact of dyslipidemia on the onset and progression of CKD,^13^ large prospective cohort studies (CRIC study, MDRD study) could not demonstrate an independent effect on disease progression in patients with established CKD.^14^^,^^15^ Some systematic reviews of randomized controlled clinical trials of adult patients with CKD suggested that lipid reduction with different agents has a beneficial effect on the decline of GFR,^12^^,^^16^ whereas others confined to statins showed that such an effect was limited to “high-intensity preparations”^17^ or absent,^18^ as clearly shown in the SHARP trial.^19^ Taken together, conflicting findings exist regarding the effect of dyslipidemia and lipid-lowering therapy, respectively, on CKD progression in adult patients.

One reason for these discrepant findings could be the use of exclusively baseline lipid levels for outcome associations in many studies, thus neglecting the impact of CKD progression on lipid levels and the intraindividual variability of lipid levels over time. To address this shortcoming, various trajectory modelling techniques have been developed.^20^

We have previously described the prevalence and pattern of dyslipidemia, as well as associated risk factors, in a large cohort of children enrolled in the Cardiovascular Comorbidity in Children with Chronic Kidney Disease (4C).^21^ Here, we have expanded this analysis to evaluate the impact of both, baseline lipid levels and longitudinal lipid trajectories, on the progression of CKD.

## Methods

### Study Setting and Design

The Cardiovascular Comorbidity in Children with Chronic Kidney Disease Study (ClinicalTrials.gov NCT01046448) prospectively observed 704 children aged 6 to 17 years with CKD stage 3 to 5 (nondialysis, no previous transplantation) and a baseline eGFR of 10 to 60 ml/min per 1.73 m^2^, who were enrolled between 2010 and 2012 at 55 pediatric nephrology units in 12 European countries and followed-up with until the end of 2018. The study was approved by the Ethics Committee of the University of Heidelberg (S-032/2009) and the institutional review boards of each participating center. Written informed consent was obtained from all parents and participants, where appropriate. Patients underwent standardized biannual clinical evaluations, including the collection of blood and urine samples, recording of fasting status, and an annual cardiovascular examination. A detailed study protocol and an analysis of the cardiovascular phenotype of this cohort has been previously published.^22^^,^^23^

### Definitions

Abnormal lipid levels were defined according to previously published cutoff levels for normal serum lipids in children (Supplementary Table S1).^24^

Primary kidney diseases were classified as CAKUT, glomerular diseases, and others.

CKD progression (expressed as kidney survival) was defined as the time to a composite event of 50% reduction in eGFR, eGFR < 10 ml/min per 1.73 m^2^, or start of kidney replacement therapy, whichever occurred first. If 50% reduction in eGFR occurred between 2 study visits, interpolation was used to determine the time point of the event.

Body mass index, height, and office BP were normalized by using the calculation of SD scores (SDS) based on reference values for healthy children.^25^, ^26^, ^27^ eGFR was calculated using the bedside approximation (0.413 × (height [cm] /serum creatinine [mg/dl]) of the updated Schwartz equation.^28^

The albumin-to-creatinine ratio in spot urine was used to quantify proteinuria as recommended by Kidney Disease: Improving Global Outcomes guidelines.^29^ There is currently no standard definition for nephrotic range albuminuria in children.^30^ A urinary protein excretion > 1 g/m^2^/d and > 3.5 g/d is the accepted cutoff for nephrotic range proteinuria in children and adults, respectively. As previously described,^21^ a urinary albumin-to-creatinine ratio of 1.1 g/g was derived from these thresholds and used as the cutoff to define nephrotic range albuminuria in children.

### Laboratory Methods

All blood and urine samples were measured by fully automated methods at a central laboratory (Synlab, Heidelberg) using stored serum samples collected at each 6-monthly visit. Urinary albumin and creatinine were measured in spot urine samples. All lipids were measured directly by enzymatic test on a Advia 2400 analyzer (Siemens, Munich, Germany).

### Statistical Analysis

Clinical and laboratory characteristics of the study population are described using mean (SD), median (interquartile range) or frequencies for continuous and categorical variables, respectively. By means of semiparametric GBTM, the evolution of the different lipid levels over time was analyzed with the aim of identifying clusters of individuals with similar trajectories. The PROC TRAJ macro in SAS (SAS Institute, Cary, NC) was used to fit a semiparametric mixture model to longitudinal data using maximum likelihood estimation. All patients, irrespective of the number of visits with observed lipid levels or intermittent missing values were included. The classification into 2 trajectory groups was pursued for all lipid trajectories to achieve a meaningful interpretation, for example, high versus normal. The shape of the trajectories was not prespecified but explored in the model by starting with a 2-group cubic trajectory model (third order of parameter estimates) and using in the final model for each trajectory group, the highest order which was significant.

For each lipid, a plot was created showing averaged estimated and observed trajectories. In addition, descriptive patient characteristics and baseline lipid levels were provided, stratified by the identified trajectory groups. Cox regression models were applied to analyze the association of trajectory groups with renal survival. Results of adjusted models for known confounders (age, sex, diagnosis, baseline eGFR, urinary albumin-to-creatinine ratio, body mass index SDS, serum albumin, diastolic BP SDS) were given with hazard ratio and 95% confidence interval for the effect on trajectory group-defined progression of CKD. A *P*-value < 0.05 was set as a threshold for statistical significance. All models were based on the same complete data set, patients with missing values in one of the confounders were not considered. Models with a slightly different confounder set (e.g., systolic vs. diastolic BP SDS) were compared by using the Akaike Information Criterion and the model with the lower Akaike Information Criterion (better fit) was defined as the final model. Furthermore, Kaplan-Meier plots comparing kidney survival according to lipid trajectory groups were presented. *P*-values of the log-rank test were given. All statistical analyses were performed in SAS (version 9.4).

## Results

### Patient Characteristics

A total of 689 patients had at least 1 lipid profile measured at enrollment; 8 patients taking lipid-lowering medication were excluded. Thus, 681 patients (90% Caucasian, *n* = 611; 65.8% boys, *n* = 448), aged 12.2 ± 3.3 years meeting the eligibility criteria, with a baseline median eGFR of 26.9 ± 11.6 ml/min per 1.73 m^2^, were included in the analysis (Supplementary Figure S1). Primary kidney diagnosis was CAKUT in 69% (*n* = 470), glomerular disease in 8.4% (*n* = 57) and other in 22.6% (*n* = 154) of patients. Mean body mass index was 18.4 ± 3.9 kg/m^2^ and 6.2% patients (*n* = 42) were obese, and 290 patients (42.6%) were treated with renin-angiotensin system inhibitors. During the time of observation (median: 5.1 [interquartile range: 5.0–6.1] years), the composite end point of CKD progression (kidney survival) was met by 399 patients (59%); kidney replacement therapy was started in 165, 159 patients had 50% loss of eGFR and 75 reached a eGFR < 10 ml/min per 1.73 m^2^.

First, we analyzed the association of CKD progression with lipid values at baseline, which have been published previously.^21^ As expected, there was no association of CKD progression with serum levels of any lipid when analyzed as a continuous variable (data not shown). Dyslipidemia, a binary variable defined according to published age-dependent cutoffs,^24^ was frequent in the cohort: CHOL in 27%, HDL-C in 32%, LDL-C in 15%, and TGs in 55% of patients, with no difference between stages of CKD.^21^ Dyslipidemia for each lipid was associated with disease progression when unadjusted (data not shown); however, when adjusted for known covariates in a Cox proportional hazard model, only increased TG levels were of borderline statistical significance (*P* = 0.0732; Table 1). Variables consistently associated with CKD progression in models for each lipid included age, a diagnosis other than CAKUT, eGFR at baseline, albuminuria (expressed as urinary albumin-to-creatinine ratio), the serum albumin level, and diastolic BP SDS.

**Table 1:** Cox proportional hazard model for progression of CKD including dyslipidemia and covariates at baseline

Variable	CHOL		LDL-C		HDL-C		TG	
	HR [95% CI]	*P*	HR [95% CI]	*P*	HR [95% CI]	*P*	HR [95% CI]	*P*
Lipid abnormality	1.04 [0.82, 1.32]	0.7732	1.06 [0.78, 1.43]	0.7297	1.11 [0.90, 1.39]	0.3294	1.22 [0.98, 1.53]	0.0732
Age, yrs	1.05 [1.02, 1.08]	0.0017	1.05 [1.02, 1.08]	0.0017	1.05 [1.02, 1.08]	0.0022	1.06 [1.03, 1.09]	0.0004
Sex (ref. male)	0.82 [0.66, 1.02]	0.0776	0.82 [0.66, 1.03]	0.0820	0.83 [0.67, 1.04]	0.1000	0.84 [0.67, 1.04]	0.1143
Other diagnosis (ref. CAKUT)	2.08 [1.63, 2.66]	< 0.0001	2.08 [1.63, 2.66]	< 0.0001	2.12 [1.66, 2.70]	<0.0001	2.11 [1.65, 2.70]	< 0.0001
Glomerular disease (ref. CAKUT)	1.29 [0.86, 1.92]	0.2151	1.28 [0.86, 1.91]	0.2275	1.36 [0.93, 1.99]	0.1179	1.28 [0.87, 1.88]	0.2062
eGFR (ml/min per 1.73 m^2^)	0.92 [0.91, 0.93]	< 0.0001	0.92 [0.91, 0.93]	< 0.0001	0.92 [0.91, 0.93]	< 0.0001	0.92 [0.91, 0.93]	< 0.0001
Log UACR	1.30 [1.20, 1.41]	< 0.0001	1.30 [1.20, 1.41]	< 0.0001	1.31 [1.21 1.42]	< 0.0001	1.30 [1.20, 1.41]	< 0.0001
BMI SDS	0.99 [0.91, 1.07]	0.7601	0.99 [0.91, 1.07]	0.7496	0.99 [0.91, 1.07]	0.7281	0.99 [0.91, 1.07]	0.7791
Serum albumin, g/l	0.93 [0.91, 0.95]	< 0.0001	0.93 [0.91, 0.95]	0.0001	0.94 [0.91, 0.96]	< 0.0001	0.93 [0.91, 0.95]	< 0.0001
Diastolic BP - SDS	1.16 [1.07, 1.27]	0.0007	1.16 [1.07, 1.27]	0.0006	1.16 [1.07, 1.27]	0.0006	1.15 [1.06, 1.25]	0.0019

BMI, body mass index; BP, blood pressure; CAKUT, congenital anomalies of the kidneys and urinary tracts; CHOL, total cholesterol; CI, confidence interval; HDL-C, high-density lipoprotein cholesterol; HR, hazard ratio; LDL-C, low-density lipoprotein cholesterol; SDS, SD scores; ref., reference category; TG, triglycerides; UACR, urinary albumin-creatinine ratio.

Variables consistently associated with progression shaded in grey.

Data were further analyzed in the subgroups of patients with (*n* = 183, 26.8%) or without (*n* = 491, 72.2%) nephrotic range proteinuria at baseline. Patients with nephrotic range proteinuria had higher baseline levels of CHOL (199 ± 62 vs. 172 ± 37 mg/dl), TG (170 ± 101 vs. 138 ± 78 mg/dl), and LDL-C (113 + 51 vs. 92 ± 30 mg/dl), whereas HDL-C levels (49 ± 16 vs. 48 ± 14 mg/dl) were similar.^21^ Kaplan-Meier survival analysis showed that patients with nephrotic range proteinuria had a much faster progression of CKD (Supplementary Figure S2), with a median kidney survival time of 1.29 (CI: 1.07–1.50) years compared with 4.76 (CI: 4.28–5.25) years for patients without nephrotic range proteinuria. To estimate whether dyslipidemia had an association with disease progression independent of nephrotic range proteinuria, a Cox proportional hazard model, including the binary variable dyslipidemia and covariates was constructed in these subgroups (Supplementary Tables S2 and S3). Hypertriglyceridemia was associated with disease progression only in patients without nephrotic range proteinuria (*P* = 0.0180). All other forms of dyslipidemia had no impact on the disease progression models for both subgroups of patients.

**Figure 1:**
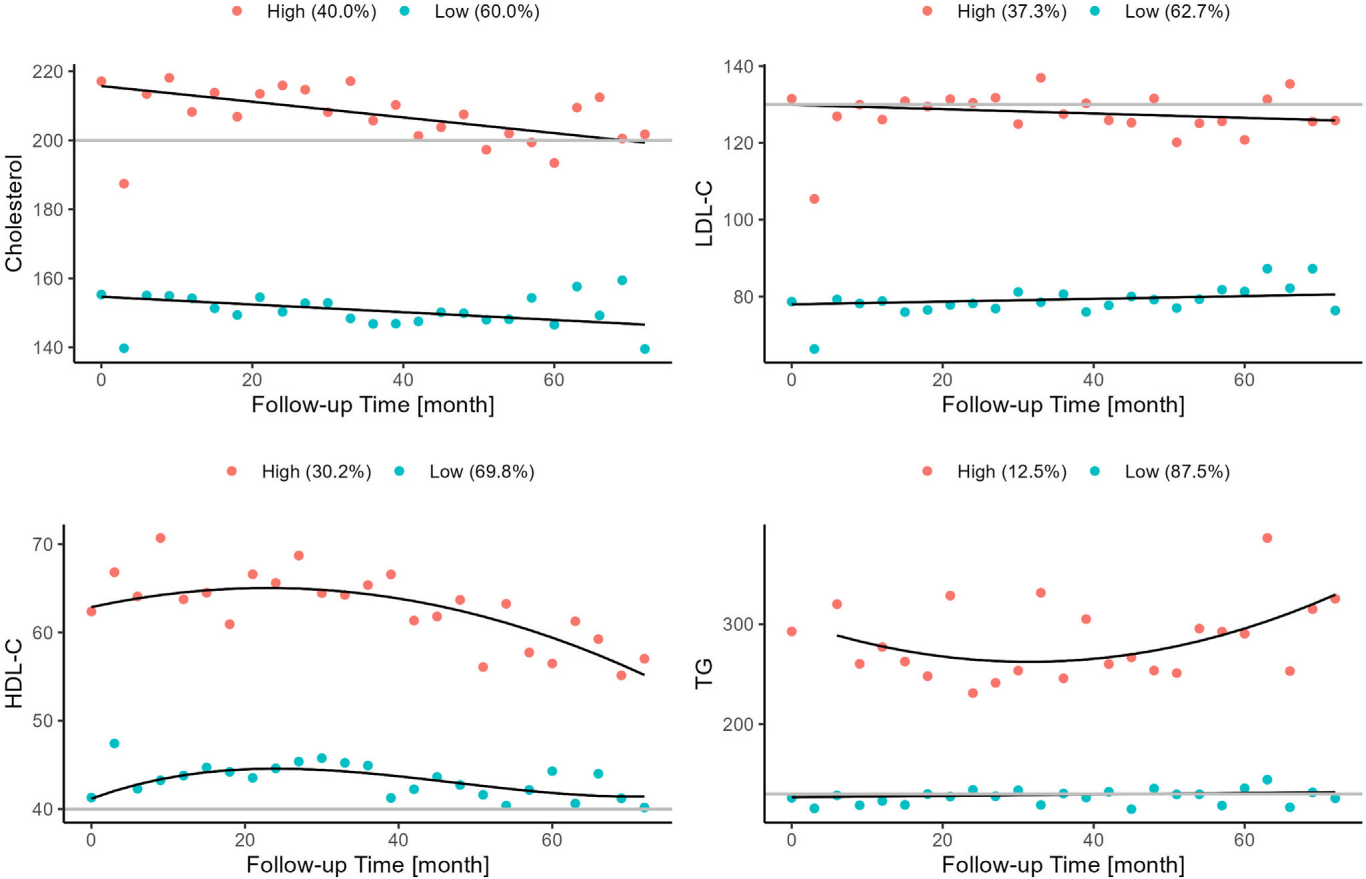
Averaged estimated (solid line) and observed (points) trajectories for each lipid. Trajectories were defined by GBTM. Classification into 2 trajectory groups with consistently high (red) versus low (blue) serum lipid levels during follow-up. Straight grey lines indicate age-related cutoffs for high and low serum levels for cholesterol and LDL-cholesterol, low levels of HDL-cholesterol and high levels of triglycerides, respectively. GBTM, group-based trajectory modeling, HDL, high-density lipoprotein; LDL, low-density lipoprotein.

Next, 2 lipid trajectories spanning an observation period of up to 8 years were created for each measured lipid based on GBTM (Figure 1). The 2 trajectories, termed “high” and low” for each lipid separated the respective lipid levels in 2 distinct groups without overlap. The high trajectory of CHOL, HDL-C, and to a lesser extent LDL-C, decreased over time. The observations contributing to the TG trajectory showed more variability, resulting in a decrease followed by an increase in the average. The low trajectories of all lipids remained rather stable.

The values contributing to the high trajectory for CHOL were clearly distributed above age-related cutoff for high CHOL (200 mg/dl), with a tendency to decrease during follow-up. The high TG trajectory values were far above the cutoff for high TG (100 or 130 mg/dl depending on age) with a U-shaped configuration of the high trajectory. The high LDL-C trajectory extended close to the age-related cutoff (130 mg/dl), whereas the low HDL-C trajectory showed most observations in an acceptable range; that is, above the age-related cutoff for low levels (40 mg/dl). Thus, most observations included in the high LDL-C trajectory correspond to age-related high values, whereas the HDL trajectories can be considered low- and high-normal age-related values.

The characteristics of all patient subgroups defined by trajectories are summarized in Table 2. Clinical and biochemical characteristics (other than lipids) were not different between trajectories; however, patients with glomerular diseases were more likely to be included in the high trajectory for CHOL, LDL-C and TG.

**Table 2:** Patient characteristics stratified by trajectory groups

Variable	CHOL low	CHOL high	LDL low	LDL high	HDL low	HDL high	TG low	TG high
*n*	409	272	433	248	479	202	599	82
Age [yrs]	12.4 (3.2)	11.8 (3.5)	12.4 (3.3)	11.7 (3.5)	12.5 (3.3)	11.4 (3.4)	12.2 (3.3)	11.9 (3.3)
% male	67.7	62.9	65.8	65.7	71.0	53.5	64.3	76.8
% Caucasian ethnicity	90.0	89.3	89.8	89.5	89.6	90.1	90.7	82.9
% glomerular disease	5.1	13.2	5.5	13.3	8.6	7.9%	6.7%	20.7
% pubertal	48.9	38.9	48.1	39.2	49.9	33.0	45.0	43.9
Time since CKD stage 2 [yrs]	6.1 (4.7)	6.0 (4.4)	6.1 (4.6)	6.1 (4.4)	5.9 (4.7)	6.5 (4.2)	6.1 (4.6)	5.7 (4.3)
Physical activity								
% none	24.7	22.6	24.2	23.1	25.1	20.9	22.9	30.9
% 1–4 h/ wk	30.4	39.8	31.3	39.3	32.7	37.8	34.1	34.6
% >4 h/ wk	44.9	37.6	44.5	37.6	42.3	41.3	43.0	34.6
Height SDS	−1.39 (1.31)	−1.28 (1.43)	−1.40 (1.30)	−1.26 (1.45)	−1.34 (1.37)	−1.37 (1.34)	−1.37 (1.36)	−1.18 (1.33)
BMI SDS	−0.03 (1.24)	0.29 (1.31)	−0.01 (1.28)	0.28 (1.25)	0.16 (1.27)	−0.06 (1.29)	0.01 (1.27)	0.74 (1.17)
% obese	4.9	8.1	5.5	7.3	6.9	4.5	4.9	15.9
Systolic BP [mmHg]	111.9 (14.2)	113.1 (15.8)	112.4 (15.0)	112.2 (14.6)	113.4 (15.4)	109.8 (13.3)	111.9 (14.4)	115.9 (17.5)
[SDS]	0.72 (1.34)	0.93 (1.36)	0.78 (1.38)	0.85 (1.30)	0.83 (1.40)	0.75 (1.22)	0.76 (1.31)	1.12 (1.58)
Diastolic BP [mmHg]	69.0 (12.0)	69.1 (13.0)	69.2 (12.3)	68.8 (12.5)	69.6 (12.6)	67.7 (11.8)	68.8 (12.0)	71.0 (14.7)
[SDS]	0.67 (1.07)	0.70 (1.10)	0.69 (1.09)	0.68 (1.07)	0.71 (1.12)	0.62 (1.00)	0.66 (1.05)	0.87 (1.27)
eGFR [ml/min per 1.73 m^2^]	27.0 (11.7)	26.7 (11.3)	26.8 (11.6)	27.1 (11.5)	26.2 (11.5)	28.5 (11.5)	27.1 (11.6)	25.3 (11.0)
UACR [g/g]	0.74 (1.05)	1.47 (2.35)	0.75 (1.17)	1.52 (2.34)	1.02 (1.70)	1.06 (1.79)	0.91 (1.43)	1.91 (3.01)
Hemoglobin [g/dl]	11.7 (1.7)	11.7 (1.6)	11.7 (1.7)	11.7 (1.6)	11.7 (1.7)	11.8 (1.4)	11.7 (1.6)	11.4 (1.7)
Bicarbonate [mmol/l)	21.1 (3.6)	21.5 (3.7)	21.1 (3.6)	21.6 (3.8)	21.2 (3.6)	21.6 (3.7)	21.2 (3.7)	21.8 (3.2)
CRP [mg/dl]	4.7 (15.6)	2.58 (5.42)	4.31 (14.92)	3.01 (6.79)	4.05 (12.6)	3.35 (12.7)	3.99 (13.3)	2.76 (5.82)
Serum albumin [g/l]	39.5 (4.5)	38.2 (6.8)	39.5 (4.5)	38.0 (6.9)	38.8 (5.8)	39.3 (5.0)	39.4 (5.0)	36.1 (8.1)
Total cholesterol (mg/dl)	154 (27)	219 (47)	157 (29)	220 (48)	175 (49)	191 (43)	174 (42)	220 (66)
HDL-cholesterol (mg/dl)	46 (13)	51 (16)	47 (15)	49 (14)	41 (9)	63 (13)	49 (14)	38 (11)
LDL-cholesterol (mg/dl)	78 (21)	128 (40)	78 (20)	133 (39)	97 (40)	101 (38)	96 (35)	117 (57)
Triglycerides (mg/dl)	130 (70)	172 (101)	134 (76)	170 (98)	161 (93)	114 (57)	127 (57)	296 (114)
RAS Inhibitor treatment (%)	40.1	46.3	40.6	46.0	42.8	42.1	40.2	59.8

BMI, body mass index; BP, blood pressure; CHOL, cholesterol; CKD, chronic kidney disease; CRP, C-reactive protein; eGFR, estimated glomerular filtration rate; HDL, high-density lipoprotein; LDL, low-density lipoprotein; RAS, renin-angiotensin system; SDS, SD scores; TG, triglycerides; UACR, urinary albumin-to-creatinine ratio.

Data expressed as mean (SD) or %

**Figure 2:**
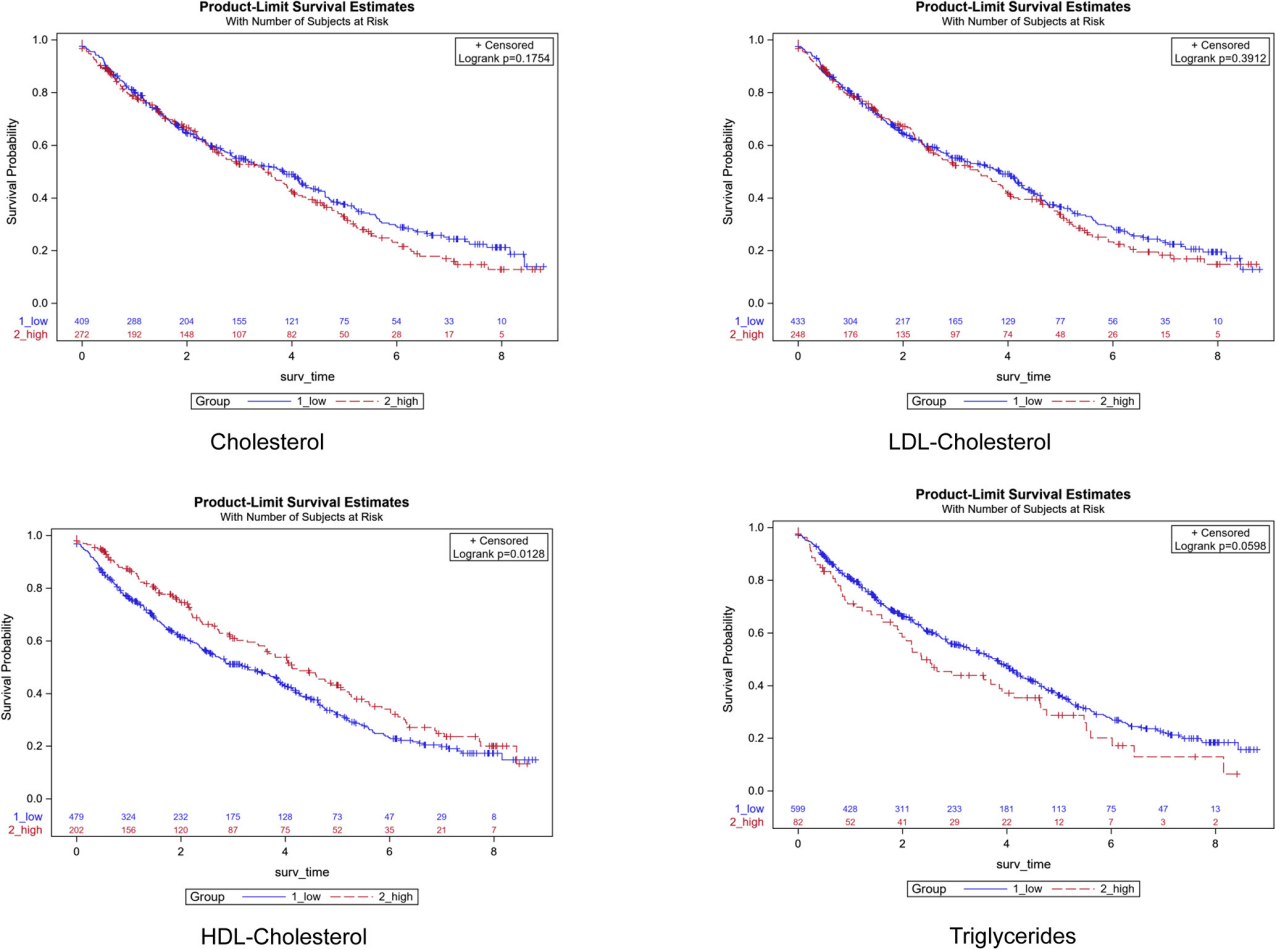
Kaplan-Maier plots of kidney survival for longitudinal serum lipid trajectories. Kidney survival was defined as the time to a composite event of 50% reduction in eGFR, eGFR <10 ml/min per 1.73 m^2^ or start of kidney replacement therapy (KRT), whichever occurred first. eGFR, estimated glomerular filtration rate.

Kaplan-Meier plots showed a difference of kidney survival for the HDL trajectory (*P* = 0.0128), but not for the TGs (*P* = 0.0503), CHOL (*P* = 0.1756), and LDL-C (*P* = 0.3912) trajectories (Figure 2). However, trajectory grouping for all lipids had no impact on disease progression when analyzed in a Cox proportional hazard analysis including covariates (Table 3). Similar to the analysis of baseline data of the study cohort (Table 1), the variables age, a diagnosis other than CAKUT, eGFR at baseline, albuminuria, serum albumin, and diastolic BP SDS were consistently associated with disease progression in the models for each lipid. Systolic and diastolic BP SDS showed the same associations in these models, but the model fit was better for the diastolic variable. Treatment with renin-angiotensin system inhibitors was more frequent in patients with a high TG trajectory (*P* < 0.001), but not in the other trajectory groups (Table 2) and was not associated with disease progression if included in the Cox proportional hazard models for all lipid trajectories (Table 3), resulting in a worse model fit (data not shown).

**Table 3:** Cox proportional hazard model for progression of CKD including lipid trajectories and covariates

Variable	CHOL		LDL-C		HDL-C		TG	
	HR [95% CI]	*P*	HR [95% CI]	*P*	HR [95% CI]	*P*	HR [95% CI]	*P*
Lipid Trajectory group (ref.low for CHOL, LDL-C and TG, ref. high for HDL-C)	1.01 [0.82, 1.25]	0.9216	0.98 [0.79, 1.22]	0.8514	1.15 [0.92, 1.45]	0.2155	1.03 [0.75, 1.41]	0.8596
Age, yrs	1.05 [1.02, 1.08]	0.0016	1.05 [1.02, 1.08]	0.0019	1.05 [1.02, 1.08]	0.0020	1.05 [1.02, 1.08]	0.0016
Sex (ref male)	0.83 [0.66, 1.03]	0.0822	0.82 [0.66, 1.03]	0.0835	0.84 [0.68, 1.05]	0.1354	0.83 [0.66, 1.03]	0.0867
Glom. disease (ref. CAKUT)	1.32 [0.90, 1.94]	0.1592	1.33 [0.91, 1.95]	0.1448	1.34 [0.92, 1.96]	0.1292	1.32 [0.89, 1.94]	0.1642
Other diagnosis (ref. CAKUT)	2.09 [1.64, 2.67]	<0.0001	2.09 [1.64, 2.67]	<0.0001	2.09 [1.64, 2.67]	<0.0001	2.09 [1.64, 2.67]	<0.0001
eGFR (ml/min per 1.73 m^2^)	0.92 [0.91, 0.93]	<0.0001	0.92 [0.91, 0.93]	<0.0001	0.92 [0.91, 0.94]	<0.0001	0.92 [0.91, 0.93]	<0.0001
Log UACR	1.31 [1.21 1.41]	<0.0001	1.31 [1.21, 1.42]	<0.0001	1.31 [1.22, 1.42]	<0.0001	1.31 [1.21, 1.42]	<0.0001
BMI-SDS	0.99 [0.91, 1.07]	0.7730	0.99 [0.91, 1.08]	0.8195	0.99 [0.91, 1.07]	0.8042	0.99 [0.91, 1.07]	0.7585
Serum Albumin	0.93 [0.91, 0.95]	<0.0001	0.93 [0.91, 0.95]	<0.0001	0.93 [0.91, 0.95]	<0.0001	0.93 [0.91, 0.95]	<0.0001
Diastolic BP -SDS	1.16 [1.07, 01.27]	0.0007	1.16 [1.07, 1.27]	0.0007	1.16 [1.06, 1.26]	0.0008	1.16 [1.07, 1.27]	0.0007

BMI, body mass index; BP, blood pressure; CAKUT, congenital anomalies of the kidneys and urinary tracts; CHOL, cholesterol; CI, confidence interval; CKD, chronic kidney disease; eGFR, estimated glomerular filtration rate; HDL-C, high-density lipoprotein cholesterol; LDL-C, low-density lipoprotein cholesterol; SDS, SD scores; TG, triglycerides; UACR, urinary albumin-to-creatinine ratio.

Finally, a Cox proportional hazard model including the binary variable lipid trajectory and covariates was constructed for each lipid in the subgroups of patients with or without nephrotic range proteinuria (Supplementary Tables S4 and S5). None of these models showed an association of the lipid trajectory group with disease progression.

## Discussion

We have investigated the association of serum lipids with CKD progression in a large long-term prospective cohort study of young patients without diabetes, age- or lifestyle-related comorbidities, or lipid-lowering medication, which is of special interest in view of the conflicting evidence derived from similar studies in adult patients. Almost 60% of patients reached the composite end point of disease progression, which was driven by well-known risk factors, including age, a diagnosis other than CAKUT, eGFR at baseline, albuminuria, BP, and serum albumin levels. However, though unadjusted serum lipid levels were associated with CKD progression, altogether, we found no impact of serum lipid levels on CKD progression, if adjusted in Cox proportional hazard models.

We first analyzed dyslipidemia at baseline and found that abnormal CHOL, LDL-C, or HDL-C were not associated with progression of CKD. However, high TG levels in patients without (but not with) nephrotic range proteinuria were associated with CKD progression (*P* = 0.02). These data suggest that the hazard “signal” is lost in patients with heavy proteinuria, caused by the dominant effect of proteinuria driving the rapid progression of CKD observed in this group, thus overriding a contribution of TGs to disease progression in these patients. Nephrotic range proteinuria, most commonly but not exclusively found in patients with glomerular disease,^21^^,^^31^ has been found in other pediatric cohort studies to greatly accelerate progression of CKD in children.^31^^,^^32^

If one assumes a linear nephrotoxic effect of lipids, the higher levels of TG, CHOL, and LDL-C in patients with nephrotic range proteinuria should contribute to their much faster CKD progression; however, the statistical models including important covariates do not permit such a conclusion. In fact, Cox proportional hazard models for this subgroup showed no association with abnormal lipid levels whereas the association of the variables, age, eGFR, serum albumin, and diastolic BP with disease progression was consistent. There were only minor differences compared with patients without nephrotic range proteinuria regarding the impact of covariates; female sex, a diagnosis other than CAKUT, eGFR, albuminuria, and serum albumin (but not age and diastolic BP SDS as in the entire cohort) were consistently included in the model. Taken together, the analysis of baseline data could not detect an independent impact of dyslipidemia on the progression of CKD in children, except for high TG levels in patients without nephrotic range proteinuria.

In the general population, plasma lipid levels show considerable intraindividual variability if analyzed repeatedly, averaging 6.1% for CHOL, 7.4% for HDL-C, 9.5% for LDL-C, and 22.6% for TGs, respectively.^33^ Intraindividual variation reflects predominantly biological rather than analytical variability in the general population .^33^^,^^34^ Thus, examining only 1 baseline lipid sample in each patient may not account for intraindividual variability, especially of TGs, and may not reflect the impact of the complete trajectory over many years. We therefore analyzed all available lipid measurements by using GBTM, which has been increasingly used to improve risk prediction in acute and chronic diseases, including CKD.^35^^,^^36^ Conceptually, trajectory modeling diminishes the effect of variation of lipid measurements and is designed to detect differences in progression in patients belonging to the thus-defined groups.^37^

Grouping of our patient population by GBTM resulted in 2 sets of patients with either a high or a low trajectory at baseline and during the entire follow-up for the respective lipid. We found that all observed trajectories showed variability of lipid levels, especially of TG. Although these fluctuations may in part be due to changes in group size, they most likely reflect the intraindividual variations in lipid levels and the impact of decreasing kidney function. Trajectories were designed irrespective of fasting state, which may have contributed to the variability of observed TG levels; however, we could previously demonstrate that fasting was not associated with any form of dyslipidemia at baseline in this cohort (including hypertriglyceridemia).^21^

The calculated trajectories indicate a decrease in CHOL and LDL-C levels during CKD progression, which has been similarly reported in adult patients^38^ and attributed to inflammation, oxidative stress, and protein-energy wasting in more advanced CKD stages.^39^ The increase in TG levels suggests a progressively impaired catabolism of TG-rich lipoproteins and this is supported by several studies showing a negative correlation of eGFR with TG levels in adult patients with CKD 40, 41, 42 and with postprandial lipolysis in children,^43^ respectively. These trajectories show remarkable similarities to those published by Tsai *et al.*, who were the first to analyze longitudinal lipid trends and adverse outcomes in adult patients with CKD using GBTM. They found that only CHOL, both at baseline and longitudinally, had an impact on CKD progression in a large observational cohort with a follow-up over 13 years.^44^ In this regard, our results are in marked contrast to the findings of Tsai *et al.*, most likely because of the advanced age (mean: 69 years at baseline) and multiple comorbidities of their patient cohort.^44^

The analysis of our baseline data indicated an association between high TG levels and disease progression in patients without (but not with) nephrotic range proteinuria, which resembles data from the CKiD study, which used the variable dyslipidemia (which included all abnormal lipid measurements).^45^ These data suggest that proteinuria is a modulator of this association in multivariate models. However, we could not confirm this finding when examining the lipid trajectories in subgroups of patients with or without nephrotic range proteinuria.

There are only a few previous studies examining the role of lipids in CKD progression in children. Disease progression was found associated with the worsening of dyslipidemia in the CKiD cohort study of American children; within-person changes of serum lipids from baseline were associated with GFR, proteinuria, and the body mass index.^46^ A prospective pediatric CKD cohort study from Korea, which did not utilize GBTM modeling, monitored CHOL levels (but not other lipids) during 8 years of follow-up. It was found that only very high (> 240 mg/dl) levels were associated with CKD progression.^47^ Although many patient characteristics are similar in these pediatric cohorts, the mean eGFR at baseline in the CKiD cohort (53 ml/min per 1.73 m^2^) and in the Korean cohort (55.7 ml/min per 1.73 m^2^) was considerably higher than in our study, which enrolled patients with more advanced CKD stages. Thus, it could be argued that the rapid progression of CKD observed in our study prevented the detection of relatively weak risk factors for CKD progression such as dyslipidemia. Although metabolic disturbances are more severe and hypertriclyceridemia more pronounced in advanced CKD stages, we cannot rule out that inclusion of patients with stage 1 to 2 CKD would lead to different conclusions regarding a contribution of lipids to the progression of CKD, which is a limitation of the study.

Whether trajectories of individual lipids are associated with CKD progression in children has not been previously investigated. Of note, several pediatric studies have shown that lipids are associated with surrogate parameters of cardiovascular disease and their progression .^48^, ^49^, ^50^, ^51^, ^52^ It thus appears that progression of cardiovascular disease could be more strongly affected by dyslipidemia than CKD progression in children; nevertheless, additional studies of CKD progression with a more detailed analysis of lipoprotein components are needed to answer this question, which seems important to address the indication for treatment with lipid-lowering agents.

Lipoprotein composition is profoundly disturbed in CKD^53^ and strongly affected by the impact of inflammation and oxidative stress in adult^54^ and pediatric^55^ patients, which could have nephrotoxic effects,^56^ but may not be adequately reflected by the measurement of the lipid component of these macromolecular complexes. It is a limitation of our study that more detailed analytics of lipoprotein composition, subclasses (such as very low-density lipoprotein remnants), or oxidative modifications, etc., could not be performed. Most of our pediatric study population was Caucasian, limiting the relevance of our findings for other age ranges and ethnic groups.

In conclusion, we could not find an association of serum lipid trajectories during up to 8 years of follow-up in the whole cohort and in subgroups with or without nephrotic range proteinuria. These data do not provide evidence for an important role for lipids in the progression of CKD in children, but we cannot rule out nephrotoxic effects of subfraction of lipoprotein classes or of lipoprotein components not measured in this study.

## Disclosure

All the authors declared no conflicting interests.

## Supplementary Material

Supplementary File (PDF) **Figure S1.** Patient flow chart. **Figure S2.** Kaplan-Maier plot of kidney survival in patients with or without nephrotic range proteinuria. **Table S1.** Reference range for lipid levels in children. **Table S2.** Cox proportional hazard model for progression of CKD including dyslipidemia and covariates at baseline in patients without nephrotic range proteinuria. **Table S3.** Cox proportional hazard model for progression of CKD including dyslipidemia and covariates at baseline in patients with nephrotic range proteinuria. **Table S4.** Cox proportional hazard model for progression of CKD including lipid trajectories and covariates in patients without nephrotic range proteinuria. **Table S5.** Cox proportional hazard model for progression of CKD including lipid trajectories and covariates in patients with nephrotic range proteinuria.

## Footnotes

Supplementary File (PDF)
**Figure S1.** Patient flow chart. **Figure S2.** Kaplan-Maier plot of kidney survival in patients with or without nephrotic range proteinuria. **Table S1.** Reference range for lipid levels in children. **Table S2.** Cox proportional hazard model for progression of CKD including dyslipidemia and covariates at baseline in patients without nephrotic range proteinuria. **Table S3.** Cox proportional hazard model for progression of CKD including dyslipidemia and covariates at baseline in patients with nephrotic range proteinuria. **Table S4.** Cox proportional hazard model for progression of CKD including lipid trajectories and covariates in patients without nephrotic range proteinuria. **Table S5.** Cox proportional hazard model for progression of CKD including lipid trajectories and covariates in patients with nephrotic range proteinuria.

## References

[1] Vaziri N.D. (2006). Dyslipidemia of chronic renal failure: The nature, mechanisms, and potential consequences. Am J Physiol Renal Physiol.

[2] Kaysen G.A., Don B., Schambelan M. (1991). Proteinuria, albumin synthesis and hyperlipidaemia in the nephrotic syndrome. Nephrol Dial Transplant.

[3] Khurana M., Silverstein D.M. (2015). Etiology and management of dyslipidemia in children with chronic kidney disease and end-stage renal disease. Pediatr Nephrol.

[4] Moradi H., Vaziri N.D. (2018). Molecular mechanisms of disorders of lipid metabolism in chronic kidney disease. Front Biosci (Landmark Ed).

[5] Saland J.M., Pierce C.B., Mitsnefes M.M. (2010). Dyslipidemia in children with chronic kidney disease. Kidney Int.

[6] Muntner P., Coresh J., Smith J.C., Eckfeldt J., Klag M.J. (2000). Plasma lipids and risk of developing renal dysfunction: The atherosclerosis risk in communities study. Kidney Int.

[7] Schaeffner E.S., Kurth T., Curhan G.C. (2003). Cholesterol and the risk of renal dysfunction in apparently healthy men. J Am Soc Nephrol.

[8] Tsuruya K., Yoshida H., Nagata M. (2014). Association of the triglycerides to high-density lipoprotein cholesterol ratio with the risk of chronic kidney disease: Analysis in a large Japanese population. Atherosclerosis.

[9] Lv S., Zhang H., Chen J. (2021). The effect of triglycerides to high-density lipoprotein cholesterol ratio on the reduction of renal function: Findings from China health and retirement longitudinal study (CHARLS). Lipids Health Dis.

[10] Weldegiorgis M., Woodward M. (2022). Elevated triglycerides and reduced high-density lipoprotein cholesterol are independently associated with the onset of advanced chronic kidney disease: A cohort study of 911,360 individuals from the United Kingdom. BMC Nephrol.

[11] Grone H.J., Walli A., Grone E. (1989). Induction of glomerulosclerosis by dietary lipids. A functional and morphologic study in the rat. Lab Invest.

[12] Fried L.F., Orchard T.J., Kasiske B.L. (2001). Effect of lipid reduction on the progression of renal disease: A meta-analysis. Kidney Int.

[13] Ruan X.Z., Varghese Z., Moorhead J.F. (2009). An update on the lipid nephrotoxicity hypothesis. Nat Rev Nephrol.

[14] Rahman M., Yang W., Akkina S. (2014). Relation of serum lipids and lipoproteins with progression of CKD: The CRIC study. Clin J Am Soc Nephrol.

[15] Chawla V., Greene T., Beck G.J. (2010). Hyperlipidemia and long-term outcomes in nondiabetic chronic kidney disease. Clin J Am Soc Nephrol.

[16] Taylor K.S., McLellan J., Verbakel J.Y. (2019). Effects of antihypertensives, lipid-modifying drugs, glycaemic control drugs and sodium bicarbonate on the progression of stages 3 and 4 chronic kidney disease in adults: A systematic review and meta-analysis. BMJ Open.

[17] Sanguankeo A., Upala S., Cheungpasitporn W., Ungprasert P., Knight E.L. (2015). Effects of statins on renal outcome in chronic kidney disease patients: A systematic review and meta-analysis. PLoS One.

[18] Su X., Zhang L., Lv J. (2016). Effect of statins on kidney disease outcomes: A systematic review and meta-analysis. Am J Kidney Dis.

[19] Haynes R., Lewis D., Emberson J. (2014). Effects of lowering LDL cholesterol on progression of kidney disease. J Am Soc Nephrol.

[20] Nguena Nguefack H.L., Page M.G., Katz J. (2020). Trajectory modelling techniques useful to epidemiological research: A comparative narrative review of approaches. Clin Epidemiol.

[21] Mencarelli F., Azukaitis K., Kirchner M. (2024). Dyslipidemia in children with chronic kidney disease-findings from the cardiovascular comorbidity in Children with Chronic Kidney Disease (4C) study. Pediatr Nephrol.

[22] Querfeld U., Anarat A., Bayazit A.K. (2010). The cardiovascular comorbidity in Children with Chronic Kidney Disease (4C) study: Objectives, design, and methodology. Clin J Am Soc Nephrol.

[23] Schaefer F., Doyon A., Azukaitis K. (2017). Cardiovascular phenotypes in children with CKD: The 4C study. Clin J Am Soc Nephrol.

[24] (2011). Expert Panel on Integrated Guidelines for Cardiovascular Health and Risk Reduction in Children and Adolescents, National Heart, Lung, and Blood Institute. Expert panel on integrated guidelines for cardiovascular health and risk reduction in children and adolescents: Summary report. Pediatrics.

[25] Bonthuis M., van Stralen K.J., Verrina E. (2012). Use of national and international growth charts for studying height in European children: Development of up-to-date European height-for-age charts. PLoS One.

[26] (2004). National High Blood Pressure Education Program working group on high blood pressure in Children and adolescents. The fourth report on the diagnosis, evaluation, and treatment of high blood pressure in children and adolescents. Pediatrics.

[27] de Onis M., Onyango A.W., Borghi E., Siyam A., Nishida C., Siekmann J. (2007). Development of a WHO growth reference for school-aged children and adolescents. Bull World Health Organ.

[28] Schwartz G.J., Munoz A., Schneider M.F. (2009). New equations to estimate GFR in children with CKD. J Am Soc Nephrol.

[29] Kidney Disease: Improving Global Outcomes CKD Work Group (2024). KDIGO 2024 Clinical Practice Guideline for the Evaluation and Management of Chronic Kidney Disease. Kidney Int.

[30] Trautmann A., Boyer O., Hodson E. (2023). IPNA clinical practice recommendations for the diagnosis and management of children with steroid-sensitive nephrotic syndrome. Pediatr Nephrol.

[31] Fathallah-Shaykh S.A., Flynn J.T., Pierce C.B. (2015). Progression of pediatric CKD of nonglomerular origin in the CKiD cohort. Clin J Am Soc Nephrol.

[32] Yang E.M., Kim J., Park E. (Published online February 23, 2024). Longitudinal progression trajectory of estimated glomerular filtration rate in children with chronic kidney disease: Results from the KNOW-Ped CKD (KoreaN cohort study for Outcomes in patients With Pediatric Chronic Kidney Disease). Kidney Res Clin Pract.

[33] Smith S.J., Cooper G.R., Myers G.L., Sampson E.J. (1993). Biological variability in concentrations of serum lipids: Sources of variation among results from published studies and composite predicted values. Clin Chem.

[34] Marcovina S.M., Gaur V.P., Albers J.J. (1994). Biological variability of cholesterol, triglyceride, low- and high-density lipoprotein cholesterol, lipoprotein(a), and apolipoproteins A-I and B. Clin Chem.

[35] Burckhardt P., Nagin D., Vijayasarathy V.P.R., Padman R. (2018). Multi-trajectory modeling to predict acute kidney injury in chronic kidney disease patients. AMIA Annu Symp Proc.

[36] Li L., Astor B.C., Lewis J. (2012). Longitudinal progression trajectory of GFR among patients with CKD. Am J Kidney Dis.

[37] Nagin D.S., Jones B.L., Elmer J. (2024). Recent advances in group-based trajectory modeling for clinical research. Annu Rev Clin Psychol.

[38] Bermudez-Lopez M., Betriu A., Valdivielso J.M., Bretones Del Pino T., Arroyo D., Fernández E. (2018). Beyond the traditional lipid parameters in chronic kidney disease. Nefrol (Engl Ed).

[39] Kwan B.C., Kronenberg F., Beddhu S., Cheung A.K. (2007). Lipoprotein metabolism and lipid management in chronic kidney disease. J Am Soc Nephrol.

[40] Nagayama D., Watanabe Y., Yamaguchi T. (2023). Relationship of serum lipid parameters with kidney function decline accompanied by systemic arterial stiffness: A retrospective cohort study. Clin Kidney J.

[41] Suh S.H., Oh T.R., Choi H.S. (2022). Serum triglycerides level is independently associated with renal outcomes in patients with non-dialysis chronic kidney disease: Results from KNOW-CKD study. Front Nutr.

[42] Wang Y., Qiu X., Lv L. (2016). Correlation between serum lipid levels and measured glomerular filtration rate in Chinese patients with chronic kidney disease. PLoS One.

[43] Saland J.M., Satlin L.M., Zalsos-Johnson J., Cremers S., Ginsberg H.N. (2016). Impaired postprandial lipemic response in chronic kidney disease. Kidney Int.

[44] Tsai C.W., Huang H.C., Chiang H.Y. (2019). Longitudinal lipid trends and adverse outcomes in patients with CKD: A 13-year observational cohort study. J Lipid Res.

[45] Warady B.A., Abraham A.G., Schwartz G.J. (2015). Predictors of rapid progression of glomerular and nonglomerular kidney disease in children and adolescents: The chronic kidney disease in children (CKiD) cohort. Am J Kidney Dis.

[46] Saland J.M., Kupferman J.C., Pierce C.B. (2019). Change in dyslipidemia with declining glomerular filtration rate and increasing proteinuria in children with CKD. Clin J Am Soc Nephrol.

[47] Baek H.S., Park M.J., Song J.Y. (2023). Association between serum total cholesterol and chronic kidney disease progression in children: Results from the KNOW-PedCKD. Pediatr Nephrol.

[48] Brady T.M., Schneider M.F., Flynn J.T. (2012). Carotid intima-media thickness in children with CKD: Results from the CKiD study. Clin J Am Soc Nephrol.

[49] Khandelwal P., Murugan V., Hari S. (2016). Dyslipidemia, carotid intima-media thickness and endothelial dysfunction in children with chronic kidney disease. Pediatr Nephrol.

[50] Kotur-Stevuljevic J., Peco-Antic A., Spasic S. (2013). Hyperlipidemia, oxidative stress, and intima media thickness in children with chronic kidney disease. Pediatr Nephrol.

[51] Azukaitis K., Kirchner M., Doyon A. (2022). Arterial stiffness and chronic kidney disease progression in children. Clin J Am Soc Nephrol.

[52] Khandelwal P., Hofstetter J., Azukaitis K. (2024). Changes in the cardiovascular risk profile in children approaching kidney replacement therapy. EClinicalmedicine.

[53] Baek J., He C., Afshinnia F., Michailidis G., Pennathur S. (2022). Lipidomic approaches to dissect dysregulated lipid metabolism in kidney disease. Nat Rev Nephrol.

[54] Speer T., Rohrer L., Blyszczuk P. (2013). Abnormal high-density lipoprotein induces endothelial dysfunction via activation of Toll-like receptor-2. Immunity.

[55] Shroff R., Speer T., Colin S. (2014). HDL in children with CKD promotes endothelial dysfunction and an abnormal vascular phenotype. J Am Soc Nephrol.

[56] Noels H., Lehrke M., Vanholder R., Jankowski J. (2021). Lipoproteins and fatty acids in chronic kidney disease: Molecular and metabolic alterations. Nat Rev Nephrol.

