# Supplementary material for: Longitudinal Lipid Trajectories and Progression of CKD in Children
Source: Kidney Int Rep. 2025 Feb 17;10(5):1393–403. doi: 10.1016/j.ekir.2025.02.007 (PMC12142784; doi:10.1016/j.ekir.2025.02.007)
Supplement: Supplementary File (PDF) — Figure S1. Patient flow chart. Figure S2. Kaplan-Maier plot of kidney survival in patients with or without nephrotic range proteinuria. Table S1. Reference range for lipid levels in children. Table S2. Cox proportional hazard model for progression of CKD including dyslipidemia and covariates at baseline in patients without nephrotic range proteinuria. Table S3. Cox proportional hazard model for progression of CKD including dyslipidemia and covariates at baseline in patients with nephrotic range proteinuria. Table S4. Cox proportional hazard model for progression of CKD including lipid trajectories and covariates in patients without nephrotic range proteinuria. Table S5. Cox proportional hazard model for progression of CKD including lipid trajectories and covariates in patients with nephrotic range proteinuria. [file mmc1.pdf]

Supplementary Material

Cardiovascular Comorbidity in Children with Chronic Kidney Disease (4C) Study Collaborators
The following principal investigators contributed to the 4C Study:

Austria: G. Cortina, Children's Hospital, Innsbruck; K. Arbeiter, University Children's Hospital, Vienna. Czech Republic: J. Dusek, University Hospital Motol, Prague. France: J. Harambat, Hôpital des Enfants, Bordeaux; B. Ranchin, Hôpital Femme Mère Enfant et Université de Lyon; M. Fischbach, A.Zalosczyk, Hôpital de Hautepierre, Strasbourg. Germany: U. Querfeld, Charité Children's Hospital, Berlin; S.Habbig, University Children's Hospital, Cologne; M. Galiano, University Children's Hospital, Erlangen; R. Büscher, University Children's Hospital, Essen; C. Gimpel, Center for Pediatrics and Adolescent Medicine, Freiburg; M. Kemper, UKE University Children's Hospital, Hamburg; A. Melk, D. Thurn, Hannover Medical School, Hannover; F. Schaefer, A. Doyon, E. Wühl, Center for Pediatrics and Adolescent Medicine, Heidelberg; M. Pohl, Center for Pediatrics and Adolescent Medicine, Jena; S. Wygoda, City Hospital St. Georg, Leipzig; N. Jeck, KfH Kidney Center for Children, Marburg; B. Kranz, University Children's Hospital, Münster; M. Wigger, Children's Hospital, Rostock. Italy: G. Montini, S. Orsola-Malpighi Hospital, Bologna; F. Lugani, Istituto Giannina Gaslini, Genova; S. Testa, Fondazione Ospedale Maggiore Policlinico, Milano; E. Vidal, Pediatric Nephrology, Dialysis & Transplant Unit, Padova; C. Matteucci, S. Picca, Ospedale Bambino Gesù, Rome. Lithuania: A. Jankauskiene, K. Azukaitis, University Children's Hospital, Vilnius. Poland: A. Zurowska, Pediatric and Adolescent Nephrology, Gdansk; D. Drodz, University Children's Hospital, Krakow; M. Tkaczyk, Polish Mothers Memorial Hospital Research Institute, Lodz; T. Urasinski, Clinic of Pediatrics, Szczecin; M. Litwin, A.Niemirska, Children's Memorial Health Institute, Warsaw; M. Szczepanska, Zabrze. Portugal: A. Texeira, Hospital Sao Joao, Porto; Serbia: A. Peco-Antic, University Children's Hospital, Belgrade. Switzerland: B.Bucher, Inselspital, Bern; G. Laube, University Children's Hospital, Zurich. Turkey: A. Anarat, A.K. Bayazit, Cukurova University, Adana; F. Yalcinkaya, University Faculty of Medicine, Ankara; E. Basin, Baskent University Faculty of Medicine, Ankara; N. Cakar, Diskapi Children's Hospital, Ankara; O. Soylemezoglu, Gazi University Hospital, Ankara; A. Duzova, Y. Bilginer, Hacettepe Medical

Faculty, Ankara; H. Erdogan, Dortcelik Children's Hospital, Bursa; O. Donmez, Uludag University, Bursa; A. Balat, University of Gaziantep; A. Kiyak, Bakirkoy Children's Hospital, Istanbul; S. Caliskan, N. Canpolat, Istanbul University Cerrahpasa Faculty of Medicine, Istanbul; C. Candan, Goztepe Educational and Research Hospital, Istanbul; M. Civilibal, Haseki Educational and Research Hospital, Istanbul; S. Emre, Istanbul Medical Faculty, Istanbul, H. Alpay, Marmara University Medical Faculty, Istanbul; G. Ozcelik, Sisli Educational and Research Hospital, Istanbul; S. Mir, B. Sözeri, Ege University Medical Faculty; Izmir; O. Yavascan, Tepecik Training and Research Hospital, Izmir; Y. Tabel, Inonu University, Malatya; P. Ertan, Celal Bayar University, Manisa; E. Yilmaz, Children's Hospital, Sanliurfa. United Kingdom: R. Shroff, Great Ormond Street Hospital, London.

Supplementary Table S1
Reference range for lipid levels in children *

Category	Low	Acceptable	Borderline-High,	High
**Total cholesterol**				
0-19 yrs	—	<170	170–199	≥200
20-24 yrs		<190	190-224	≥225
**LDL cholesterol**				
0-19 yrs	—	<110	110–129	≥130
20-24 yrs		<120	120-159	≥160
**Triglycerides**				
0–9 yrs	—	<75	75–99	≥100
10–19 yrs	—	<90	90–129	≥130
20-24 yrs		<115	115-149	≥150
**HDL cholesterol**				
0-24 yrs	<40	>45	40-45	—

* All in mg/dl.
" Expert panel on integrated guidelines for cardiovascular health and risk reduction in children and adolescents. Summary report", Pediatrics 2011; 128: S213–S256

Supplementary Table S2

Cox proportional hazard model for progression of CKD including dyslipidemia and covariates at baseline in patients without nephrotic range proteinuria

	CHOL *N=485*		LDL-C *N=484*		HDL-C *N=487*		TG *N=486*	
	HR [95%CI]	*P*	*HR [95%CI]*	*P*	*HR [95%CI]*	*P*	*HR [95%CI]*	*P*
Lipid abnormality	0.93 [0.68, 1.28	0.6596	1.14 [0.74, 1.75]	0.5525	1.21 [0.92, 1.59]	0.1179	1.39 [1.06, 1.84]	0.0180
Age, yrs	1.03 [0.99, 1.07]	0.0955	1.00 [1.00, 1.08]	0.0713	1.03 [0.99, 1.07]	0.1198	1.05 [1.01, 1.10]	0.0172
Sex (ref male)	0.67 [0.51, 0.89]	0.0065	0.67 [0.51, 0.90]	0.0075	0.69 [0.51, 0.91]	0.0099	0.70 [0.52, 0.93]	0.0146
Glomerular disease (ref CAKUT)	1.19 [060, 2.56]	0.6233	1.11 [0.55, 2.22]	0.7734	1.28 [0.67, 2.44]	0.4543	1.18 [0.62, 2.24]	0.6179
Other diagnosis (ref CAKUT)	2.60 [1.92, 3.52]	<0.0001	2.61 [1.93, 3.53]	<0.0001	2.59 [1.92, 3.51]	<0.0001	2.58 [1.91, 3.50]	< 0.0001
eGFR (ml/min/1.73 m^2)	0.92 [0.91, 0.94]	<0.0001	0.92 [0.91, 0.94]	<0.0001	0.92 [0.91, 0.94]	<0.0001	0.92 [0.91, 0.94]	<0.0002
Log UACR	1.23 [1.11, 1.36]	<0.0001	1.22 [1.10, 1.35]	0.0001	1.23 [1.11, 1.36]	<0.0001	1.22 [1.10, 1.35]	0.0001
BMI SDS	1.04 [0.94, 1.16]	0.4250	1.04 [0.93, 1.15]	0.5166	1.04 [0.93, 1.15]	0.5210	1.04 [0.94, 1.15]	0.4482
Serum albumin, g/L	0.93 [0.90, 0.97]	0.0003	0.93 [0.89, 0.97]	0.0002	0.94 [0.90, 0.97]	0.0007	0.93 [0.90, 0.97]	0.0002
Diastolic BP - SDS	1.12 [1.00, 1.26]	0.0603	1.11 [0.99, 1.25]	0.0702	1.21 [1.00, 1.26]	0.0551	1.09 [0.97, 1.23]	0.1361

HR = hazard ratio, CI = confidence interval, ref = reference category, UACR=urinary albumin-creatinine ratio

Supplementary Table S3

Cox proportional hazard model for progression of CKD including dyslipidemia and covariates at baseline in patients with nephrotic range proteinuria

	CHOL *N=183*		LDL-C *N=183*		HDL-C *N=183*		TG *N=183*	
	HR [95%CI]	*P*	*HR [95%CI]*	*P*	*HR [95%CI]*	*P*	*HR [95%CI]*	*P*
Lipid abnormality	1.36 [0.90, 2.05]	0.1424	0.90 [0.57, 1.43]	0.6623	0.89 [0.62, 1.29]	0.5463	0.85 [0.57, 1.26]	0.4159
Age, yrs	1.10 [1.04, 1.16]	0.0005	1.09 [1.04, 1.15]	0.0011	1.10 [1.04, 1.15]	0.0009	1.09 [1.03, 1.15]	0.0040
Sex (ref male)	1.37 [0.93, 2.02]	0.1077	1.48 [1.01, 2.16]	0.0438	1.45 [0.99, 2.11]	0.0551	1.45 [1.00, 2.11]	0.0522
Glomerular disease (ref CAKUT)	0.91 [0.53, 1.59]	0.7481	1.00 [0.57, 1.74]	0.9945	0.96 [0.56, 1.67]	0.8973	1.02 [0.58, 1.77]	0.9576
Other diagnosis (ref CAKUT)	1.38 [0.86, 2.22]	0.1765	1.39 [0.86, 2.23]	0.1771	1.38 [0.86, 2.21]	0.1857	1.36 [0.84, 2.19]	0.2091
eGFR (ml/min/1.73 m^2)	0.91 [0.86, 0.93]	<0.0001	0.91 [0.89, 0.94]	<0.0001	0.91 [0.89, 0.93]	<0.0001	0.91 [0.89, 0.94]	<0.0001
Log UACR	1.13 [0.75, 1.70]	0.5565	1.30 [0.87, 1.94]	0.2040	1.24 [0.84, 1.82]	0.2741	1.29 [0.88, 1.86]	0.1962
BMI SDS	0.86 [0.74, 1.00]	0.0505	0.87 [0.76, 1.01]	0.0744	0.88 [0.76, 1.02]	0.0778	0.88 [0.76, 1.02]	0.0925
Serum albumin, g/L	0.92 [0.87, 0.95]	<0.0001	0.92 [0.90, 0.95]	<0.0001	0.92 [0.88, 0.95]	<0.0001	0.92 [0.89, 0.95]	<0.0001
Diastolic BP - SDS	1.35 [1.16 1.57]	<0.0001	1.33 [1.14, 1.54]	0.0002	1.34 [1.15, 1.56]	0.0001	1.34 [1.16, 1.56]	0.0001

Supplementary Table S4

Cox proportional hazard model for progression of CKD including lipid trajectories and covariates in patients without nephrotic range proteinuria

	CHOL		LDL-C		HDL-C		TG	
	HR [95%CI]	*P*	*HR [95%CI]*	*P*	*HR [95%CI]*	*P*	*HR [95%CI]*	*P*
Lipid Trajectory group (ref.low for Chol, LDL-C and TG, ref. high forHDL-C)	0.98 [0.75, 1.28]	0.8774	0.99 [0.75, 1.31]	0.9456	1.22 [0.91, 1.63]	0.1847	1.26 [0.84, 1.88]	0.2686
Age, yrs	1.03 [1.00, 1.08]	0.0858	1.04 [0.96, 1.08]	0.0857	1.03 [0.99, 1.07]	0.0948	1.04 [1.00, 1.08]	0.0753
Sex (ref male)	0.68 [0.51, 0.90]	0.0078	0.68 [0.51, 0.90]	0.0075	0.70 [0.52, 0.93]	0.0144	0.68 [0.51, 0.91]	0.0087
Glom. disease (ref CAKUT)	1.23 [0.64, 2.35]	0.5306	1.23 [0.64, 2.33]	0.5374	1.21 [0.64, 2.30]	0.5581	1.19 [0.63, 2.27]	0.5962
Other diagnosis (ref CAKUT)	2.59 [1.92, 3.51]	<0.0001	2.60 [1.91, 3.51]	<0.0001	2.55[1.90, 3.46]	<0.0001	2.61[1.93, 3.53]	<0.0001
eGFR (ml/min/1.73 m^2)	0.92 [0.91, 0.94]	<0.0001	0.92 [0.91, 0.94]	<0.0001	0.92 [0.91, 0.94]	<0.0001	0.92 [0.91, 0.94]	<0.0001
Log albuminuria	1.22 [1.11 1.36]	<0.0001	1.22 [1.10, 1.36]	0.0001	1.23 [1.11, 1.37]	<0.0001	1.23 [1.11, 1.36]	<0.0001
BMI-SDS	1.04 [0.94, 1.16]	0.4584	1.04 [0.94, 1.16]	0.4666	1.04 [0.94, 1.16]	0.4345	1.03 [0.93, 1.14]	0.6103
Serum Albumin	0.93 [0.90, 0.97]	0.0003	0.93 [0.90, 0.97]	0.0003	0.93 [0.90, 0.97]	<0.0001	0.93 [0.90, 0.97]	0.0002
Diastolic BP -SDS	1.11 [1.00, 01.25]	0.0698	1.11 [0.99, 1.25]	0.0702	1.11 [0.99, 1.25]	0.0795	1.12 [0.99, 1.25]	0.0630

Supplementary Table S5

Cox proportional hazard model for progression of CKD including lipid trajectories and covariates in patients <u>with</u> nephrotic range proteinuria.

	**CHOL**		**LDL-C**		**HDL-C**		**TG**	
	HR [95%CI]	*P-value*	*HR [95%CI]*	*P value*	*HR [95%CI]*	*P value*	*HR [95%CI]*	*P value*
Lipid Trajectory group (ref. low for Chol, LDL-C and TG, ref. high forHDL-C)	1.30 [0.88, 1.93]	0.1904	1.06 [0.71, 1.57]	0.7903	0.86 [0.58, 1.30]	0.4788	0.69 [0.42, 1.15]	0.1529
Age, yrs	1.10 [1.05, 1.17]	0.0004	1.10 [1.04, 1.16]	0.0011	1.10 [1.04, 1.16]	0.0007	1.1 [1.03, 1.15]	0.0030
Sex (ref male)	1.39 [0.94, 2.04]	0.0965	1.46 [1.00, 2.13]	0.0521	1.44 [0.98, 2.10]	0.0616	1.43 [1.00, 2.08]	0.0627
Glom. disease (ref CAKUT)	0.95 [0.55, 1.64]	0.8398	0.97 [0.56, 1.69]	0.9051	0.94 [0.54, 1.65]	0.8234	1.07 [0.61, 1.88]	0.8020
Other diagnosis (ref CAKUT)	1.36 [0.84, 2.18]	0.2077	1.38 [0.86, 2.23]	0.1834	1.35 [0.84, 2.19]	0.2186	1.42 [0.89, 2.29]	0.1450
eGFR (ml/min/1.73 m^2)	0.91 [0.89, 0.93]	<0.0001	0.91 [0.89, 0.93]	<0.0001	0.91 [0.89, 0.93]	<0.0001	0.91 [0.89, 0.93]	<0.0001
Log albuminuria	1.16 [0.78 1.73]	0.4691	1.24 [0.82, 1.85]	0.3073	1.24 [0.85, 1.81]	0.2714	1.28 [0.88, 1.88]	0.1979
BMI SDS	0.86 [0.74, 1.00]	0.0452	0.87 [0.75, 1.01]	0.0692	0.88 [0.76, 1.02]	0.0798	0.89 [0.77, 1.03]	0.1104
Serum albumin	0.92 [0.90, 0.95]	<0.0001	0.92 [0.90, 0.95]	<0.0001	0.92 [0.89, 0.95]	<0.0001	0.92 [0.89, 0.95]	<0.0001
Diastolic BP -SDS	1.35 [1.17, 1.57]	<0.0001	1.34 [1.15, 1.56]	0.0002	1.33 [1.15, 1.55]	0.0002	1.37 [1.18, 1.60]	<0.0001

Supplementary Figure S1

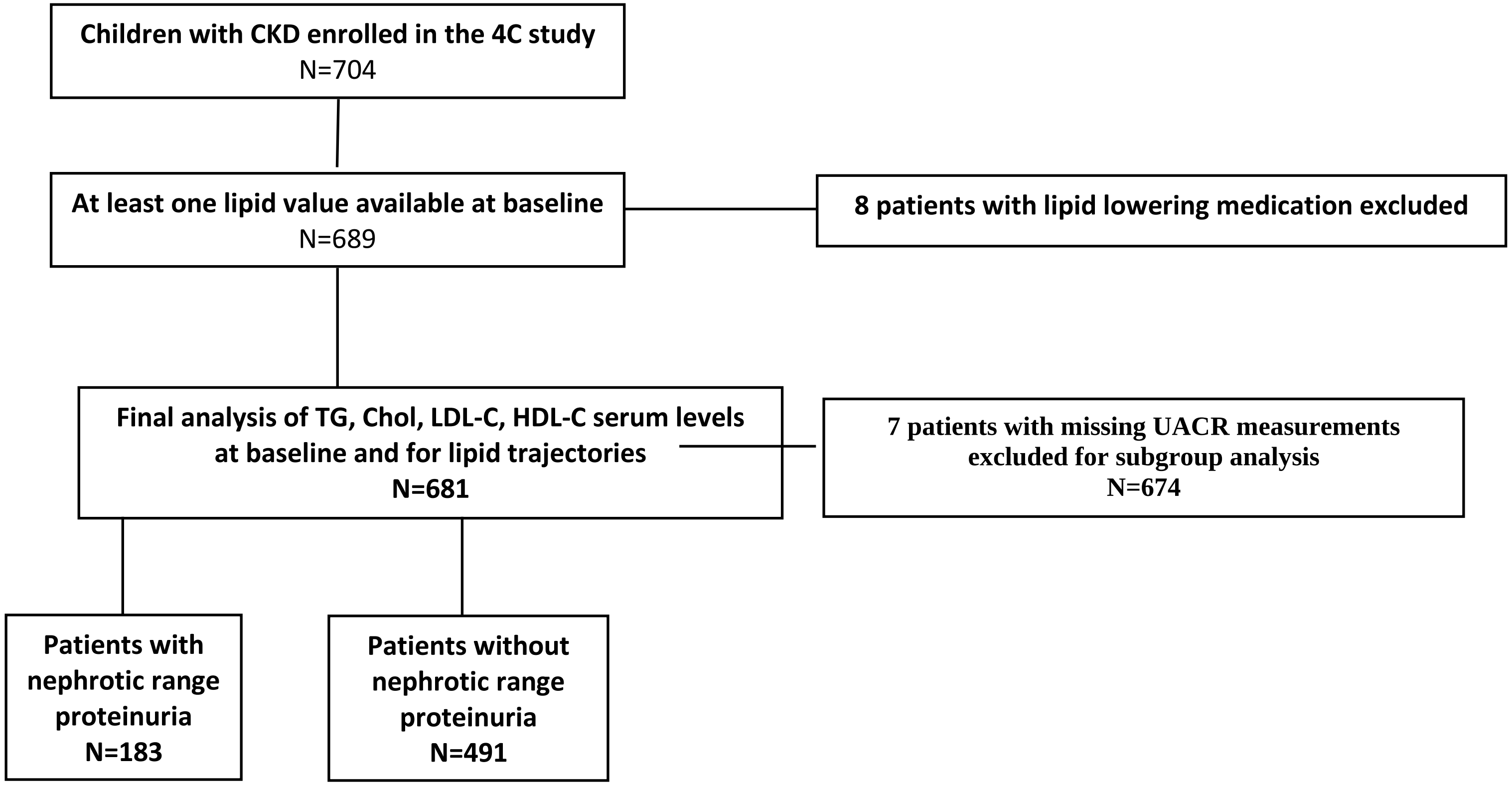

Patient Flow Chart

Supplementary Figure S2

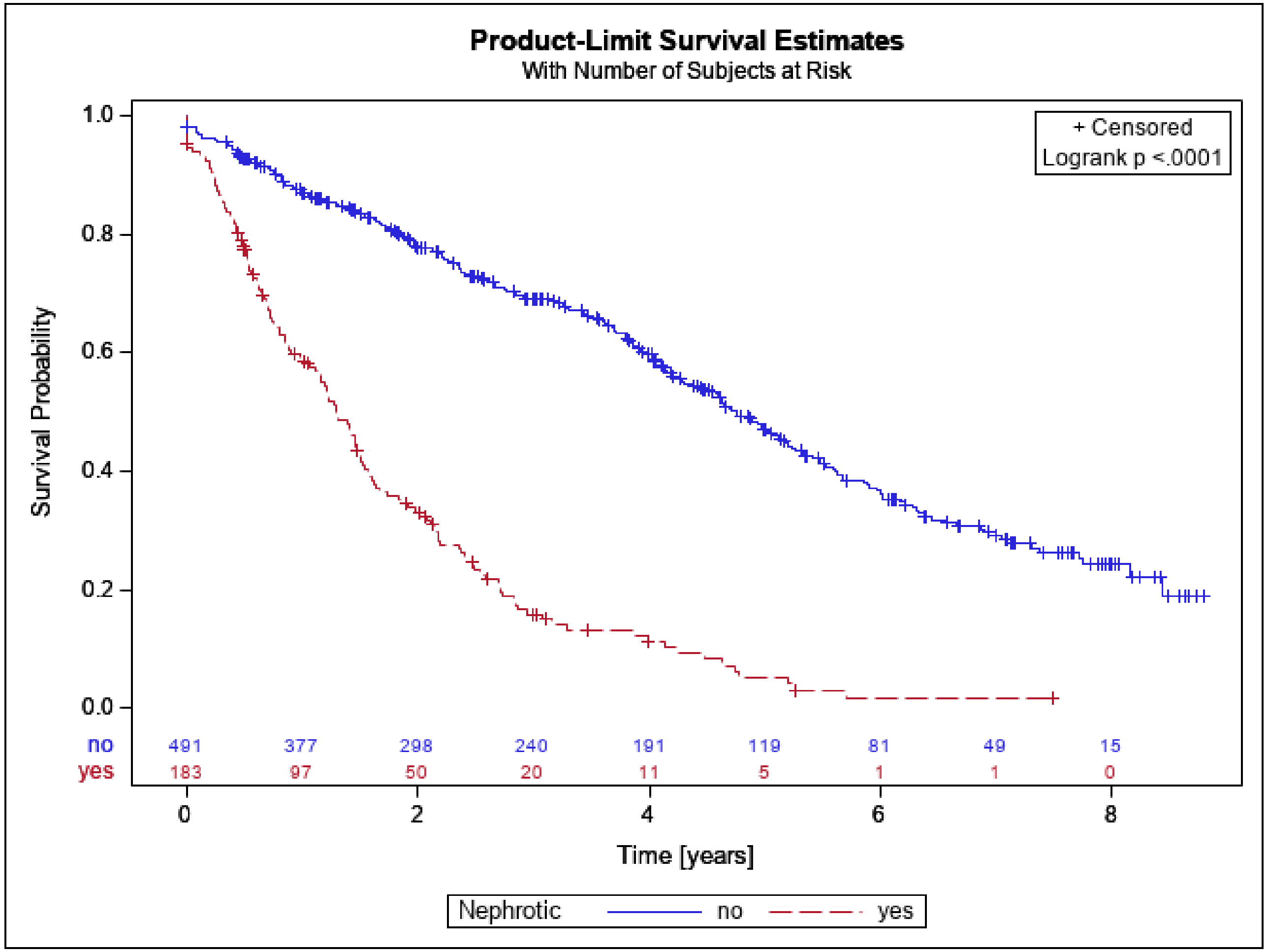

Kaplan-Maier plot of kidney survival in patients with or without nephrotic range proteinuria
